# HtrA4 Protease Promotes Chemotherapeutic-Dependent Cancer Cell Death

**DOI:** 10.3390/cells8101112

**Published:** 2019-09-20

**Authors:** Tomasz Wenta, Michal Rychlowski, Miroslaw Jarzab, Barbara Lipinska

**Affiliations:** 1 Department of General and Medical Biochemistry, Faculty of Biology, University of Gdansk, Wita Stwosza 59, 80-308 Gdansk, Poland; miroslaw.jarzab@biol.ug.edu.pl (M.J.); barbara.lipinska@biol.ug.edu.pl (B.L.); 2 Laboratory of Virus Molecular Biology, Intercollegiate Faculty of Biotechnology, University of Gdansk and Medical University of Gdansk, Abrahama 58, 80-307 Gdansk, Poland; michal.rychlowski@biotech.ug.edu.pl

**Keywords:** HtrA proteins, HtrA4 protease, apoptosis, oncogenesis, cancer

## Abstract

The HtrA4 human protease is crucial in placentation and embryo implantation, and its altered level is connected with pre-eclampsia. The meta-analyses of microarray assays revealed that the HtrA4 level is changed in brain tumors and breast and prostate cancers, which suggests its involvement in oncogenesis. In spite of the HtrA4 involvement in important physiological and pathological processes, its function in the cell is poorly understood. In this work, using lung and breast cancer cell lines, we showed for the first time that the full-length HtrA4 and its N-terminally deleted variant promote cancer cell death induced by chemotherapeutic drugs by enhancing apoptosis. The effect is dependent on the HtrA4 proteolytic activity, and the N-terminally deleted HtrA4 is more efficient in the cell death stimulation. Furthermore, HtrA4 increases the effect of chemotherapeutics on the clonogenic potential and motility of cancer cells, and it increases cell cycle arrest at the G2/M phase. HtrA4 may modulate cell death by degrading the anti-apoptotic XIAP protein and also by proteolysis of the executioner pro-caspase 7 and cytoskeletal proteins, actin and β-tubulin. These findings provide new insight into the mechanism of the HtrA4 protease function in cell death and oncogenesis, and they may help to develop new anti-cancer therapeutic strategies.

## 1. Introduction

Cancer is the leading reason for human death before age 70 years in most countries of the world. Every year the number of new cases increases, and in 2018 over 18 million new cancer cases and almost 10 million cancer deaths were diagnosed [1]. These data show that there is a necessity of developing new strategies to fight with this disease as well as improving the currently used therapies. 

HtrA4 is one of the four human HtrA proteins that are involved in maintaining cellular homeostasis. The HtrA1–3 proteases function as a protein control guard by removing misfolded and aberrant proteins to avoid the accumulation of toxic aggregates [2,3,4]. Moreover, the HtrA1–3 play an important role as pro-apoptotic proteases, which induce cell death mainly by interaction with the X-linked inhibitor of apoptosis protein (XIAP) [5,6,7,8,9,10,11]. It was observed that changed levels of these proteins were connected with neurodegenerative disorders and cancer whose development is closely connected to cell death by apoptosis [3,4]. HtrA1/3 and HtrA4 share a similar domain organization. At the N-terminus, they contain a signal peptide characteristic of the secreted proteins, followed by a domain with homology to the insulin-like growth factor binding proteins, a Kazal-type serine protease inhibitor motif, a protease domain of the chymotrypsin-type with a catalytic triad (His-Asp-Ser) and one PDZ (postsynaptic density protein 95, *Drosophila* disc large tumor suppressor and zonula occludens-1 protein domain) domain. In the HtrA4 protein, the catalytic triad is composed of His218, Asp248, and Ser326 [4,12]. Human HtrA1–3, with the exception of the HtrA3S protein, are trimeric, and structural elements of their N-terminal domains participate in oligomerization [12,13,14,15]. Recently, the in silico analysis of HtrA4 in comparison to other human HtrAs and a molecular dynamics simulation of HtrA4 in complex with a generic substrate of HtrAs (β-casein) were performed showing the importance of the N-terminal residues in oligomerization of HtrA4 [12]. Nevertheless, knowledge regarding the HtrA4 biochemical features and its physiological substrates is very limited. 

It has been shown that HtrA4 plays a crucial role in embryo implantation and placentation, and it can be a prognostic marker for pre-eclampsia since the level of this protein is significantly elevated at the early onset of pre-eclampsia [16,17,18,19,20]. On the other hand, meta-analyses of available microarray data showed changed levels of the *HtrA4* gene expression in brain tumors and the breast and prostate cancers in comparison to normal tissues, which suggests HtrA4 connection with carcinogenesis [21]. Specifically, it was shown that *HtrA4* is upregulated in glioblastoma multiforme compared to control brain from epilepsy patients and in breast carcinoma compared to normal breast samples [22,23]. On the other hand, studies by Varambally et al. [24] demonstrated that *HtrA4* is down-regulated in hormone refractory metastatic prostate cancer compared to primary prostate carcinoma. Moreover, it was indicated that the allelic ratio of *HtrA4* is lower in glioblastoma compared to normal DNA control [25]. Recently, the correlation of HtrA4 with cell proliferation and cell cycle modulation was demonstrated [26]. In spite of HtrA4 involvement in important physiological, pathological, and cellular processes, its function in the cell is poorly understood. However, the high similarity of the HtrA4 protein domain structure to that of HtrA1 and HtrA3, and implication of HtrA4 in oncogenesis suggest that it may function similarly to HtrA1/3 in promoting cancer cell death. 

In this work, we show for the first time that HtrA4 promotes death of cancer cells treated with chemotherapeutic drugs, mainly via apoptosis. We found that HtrA4 reduced survival, clonogenic potential, and motility of cancer cells. Furthermore, HtrA4 enhanced the activity of drugs routinely used in chemotherapy by modulation of the cell cycle. Our results provide new insight into the mechanism of HtrA4 action in cell death and oncogenesis. We believe that they can be helpful in developing new, complementary strategies for cancer therapy. 

## 2. Materials and Methods

### 2.1. Materials

Restriction enzymes and T4 ligase were purchased from Fermentas (Vilnius, Lithuania). Primers used in site-directed mutagenesis were purchased from Genomed S.A. (Warszawa, Poland) or Sigma-Aldrich (Poznan, Poland). Other chemicals, unless otherwise stated, were from Sigma-Aldrich. The following antibodies were obtained from Sigma: HRP-conjugated mouse monoclonal anti-β-actin (A3854) and mouse polyclonal anti-HtrA4 (SAB1402047). Rabbit polyclonal antibodies against HtrA4 (PA5-60992), rabbit polyclonal antibodies against XIAP (PA5-29253), mouse monoclonal antibodies against GAPDH (MA5-15738), mouse monoclonal antibodies against β-tubulin (MA5-16308), and mouse monoclonal antibodies against caspase 7 (MA5-15159) were obtained from Thermo Fisher Scientific (Rockford, IL, USA). 

### 2.2. Plasmid Construction

The plasmids that were used are listed in Table S1. The pTW_H4/pTW_∆H4 plasmids contained the DNA fragment encoding HtrA4 (amino acids 1–476) and ΔN-HtrA4 (amino acids 147–476), respectively, cloned into the *BamH*I and *Mlu*I sites of the pRetroX-Tight pur vector (Clontech, Inc., Mountain View, CA, USA). The pEGFP N1 vector (Clontech, Inc., Mountain View, CA, USA) was used as the source of the *EGFP* gene in further PCR-based cloning into the retroviral plasmids pBabe puro (Cell Biolabs, Inc., San Diego, CA, USA). The pTW_H4-GFP/pTW_ ∆H4-GFP plasmids contained the DNA fragment encoding HtrA4 (amino acids 1–476) fused with EGFP and ΔN-HtrA4 (amino acids 147–476) fused with EGFP respectively, cloned into the *BamH*I and *Sal*I sites of the pBabe puro vector. Mutations in the *HtrA4* genes were introduced by site-directed mutagenesis according to the protocol of the Quick-Change Mutagenesis Kit (Stratagene, La Jolla, CA, USA) as described in [27]. For interference of RNA expression, the pMKO.1 puro (gift from Bob Weinberg; Addgene # 8452) [28] containing the 5′-AAGCTACATACCCAGCCCTCCCTCGAGGGAGGGCTGGGTATGTAGCTTTTTTT-3′ *HtrA4* shRNA sequence was used. Construction of other plasmids was described in [11,27].

### 2.3. Cell Lines and Cell Culture

The human lung adenocarcinoma A549 and HCC827 cells were obtained from Sigma-Aldrich and Deutsche Sammlung von Mikroorganismen und Zellkulturen, Germany, respectively. The human breast cancer MCF7, T47D, SKBR3, BT474, and MDA-MB-231 and prostate adenocarcinoma PC-3 cell lines were from the collection of the Department of Biology and Medical Genetic, University of Gdansk, Poland. The Immortalized Ovarian Surface Epithelial IOSE364 cell line was kindly supplied by Dr. Nelly Auersperg, from the Department of Obstetrics & Gynecology, University of British Columbia, via Professor P. Dziegiel (Department of Histology and Embryology, Medical University of Wroclaw, Poland); the human bronchial epithelial cell line BEAS-2B was supplied by Professor R. Olszanecki (Department of Pharmacology, Jagiellonian University, Poland); and placental choriocarcinoma cells BeWo were provided by Professor E. Gregoraszczuk (Department of Physiology and Toxicology of Reproduction, Institute of Zoology and Biomedical Research, Jagiellonian University in Kraków, Kraków, Poland). The A549 and MDA-MB-231 cells were maintained in DMEM medium (Gibco, Paisley, UK) and Leibovitz’s L-15 medium (Gibco, Paisley, UK), respectively, containing 10% fetal bovine serum (FBS; Gibco, Paisley, UK), penicillin (100 units/mL) and streptomycin (100 μg/mL). The MCF7, BT474, SKBR3, T47D, PC-3, and HCC827 cells were grown in RMPI 1640 medium (Gibco, Paisley, UK) supplemented with 10% or 20% fetal bovine serum (Gibco, Paisley, UK) and standard antibiotics. The BEAS-2B and IOSE364 cells were cultured in bronchial epithelial cell growth medium (Lonza, Walkersville, MD, USA) without gentamycin and in M199/MCDB105 1:1 medium containing 10% FBS and 2 mM l-glutamine, respectively. The MDA-MB-231 and BeWo cells were grown in Leibovitz’s L-15 medium (Gibco, Paisley, UK) and in F-12K Medium (Gibco, Paisley, UK), respectively, supplemented with 10% fetal bovine serum (Gibco, Paisley, UK) and standard antibiotics. The cells were maintained at 37 °C in a humidified atmosphere with 5% CO_2_. 

### 2.4. Western Blotting

Cells grown to 80–90% of confluency were homogenized on ice with the RIPA buffer: 50 mM Tris-HCl pH 8.0, 150 mM NaCl, 0.1% SDS, 1% Triton X-100, 0.5% sodium deoxycholate containing 2 mM PMSF (phenylmethylsulfonyl fluoride), 10 μg/mL aprotinin, and 10 μg/mL leupeptin. Samples were then cleared by centrifugation at 14,000× *g* for 30 min at 4 °C, and the protein concentration was estimated by staining with amido black as described previously [27]. Samples containing equal amounts of protein were resolved by SDS-PAGE and then transferred onto Immobilon membrane. The membranes were incubated for 1 h in 4% skimmed milk and probed with specific primary antibodies overnight at 4 °C. Secondary antibodies conjugated with HRP and Clarity Western ECL Substrate (BIO-RAD, Hercules, CA, USA) were used to visualize specific protein bands. The bands were detected using the Azure Biosystems Inc., Bioanalytical Imaging System, model c400 (USA).

### 2.5. Retroviral Transduction

Viral particles were produced by co-transfecting 10 μg of the pTW_H4-GFP or pTW_∆H4-GFP plasmid, based on pBabe puro (Clontech, Inc., Mountain View, CA, USA), and 5 μg of the envelope protein-expressing plasmid pCMV-VSV-G (Cell Biolabs, Inc., San Diego, CA, USA) into packaging cells GP2-293 (Clontech, Inc., Mountain View, CA, USA), using CalPhos™ Mammalian Transfection Kit (Clontech, Inc., Mountain View, CA, USA), according to the manufacturer’s protocol as described in [10]. To obtain cells with inducible HtrA4 expression, the Retro-X™ Tet-On Advanced Inducible Expression System (Clontech, Inc., Mountain View, CA, USA) was used. Viral particles were produced by co-transfecting 10 μg of the pTW2_H4, pTW2_H4Q, pTW2_∆H4, or pTW2_∆H4Q plasmid (encoding the HtrA4, HtrA4S326A, ΔN-HtrA4 or ΔN-HtrA4 S326A, respectively) and 5 μg of the pCMV-VSV-G plasmid into the packaging GP2-293 cells, as described in [27]. A stable A549 cell line (obtained as described above), constitutively expressing the tetracycline-controlled transactivator rtTA-Advanced, was then transduced with the viral particles. The transduced cells were selected with 750 μg/mL of G418 (geneticin) (Gibco, Paisley, UK). The selected cells were cultured at least for 15 days in the medium supplemented with 1 μg/mL puromycin. The presence of the exogenous proteins was tested by fluorescence microscopy and western blotting. In a similar way, using the pMKO1sh4 and pCMV-VSV-G plasmids, the cells with suppression of *HtrA4* were obtained. As a control, we used the cells transfected with empty vector pMKO1 puro and pCMV-VSV-G. The interference efficiency was tested by western blotting.

### 2.6. Fluorescence Microscopy

The MDA-MB-231, MCF7, and T47D cells with endogenous production of HtrA4 were seeded on coverslips (16 × 16 mm, Marienfeld, Germany) and allowed to grow overnight. The cells were fixed in PBS, containing 4% formaldehyde, for 10 min at room temperature. Next, the coverslips were placed on ice, and the cells were permeabilized by adding 0.2% Triton-X100 in PBS for 5 min before blocking with a PBSB solution (i.e., PBS containing 4% BSA) for 60 min. After blocking, the cells were incubated with the anti-HtrA4 rabbit IgG (1:87) in PBSB for 1 h. Next, the cells were washed three times with PBS and incubated for 1 h with the secondary anti-rabbit antibodies (1:2500) conjugated with Alexa Fluor 488. 

The A549 cells expressing HtrA4/ΔN-HtrA4-GFP were observed alive using fluorescent properties of GFP. Mitochondria were labeled for 30 min by adding 150 nM Mito RED. Specimens were imaged using a confocal laser scanning microscope (Leica SP8X) with a 63× oil immersion lens (Leica, Wetzlar, Germany). The analyses of the HtrA4 and partner protein co-localizations were performed using the LAS AF 3.3.0 software. The pixel intensities were quantified and are presented as Pearson’s correlation and overlap coefficient.

### 2.7. Cell Viability Assay

The A549 cells with inducible expressions of HtrA4, HtrA4 S326A, ΔN-HtrA4, or ΔN-HtrA4 S326A were seeded at a density of 3 × 10^3^ cells per well in a 96-well plate and allowed to grow overnight. To induce expression of a *HtrA4*, 1 μg/mL of doxycycline was added to the medium 24 h before treatment with etoposide. The cells were treated with increasing concentrations of etoposide (0–310 μM) for 48 h. Control samples were treated with increasing concentrations of etoposide but without doxycycline. A similar procedure was used with the cells with suppressed *HtrA4* expression. For the MCF7 cells, increasing concentrations of cisplatin were used (0–1 mM). In the MTT assay, 25 μL of MTT (3-(4,5-dimethylthiazol-2-yl)-2,5-diphenyltetrazolium bromide) stock solution (4 mg/mL in PBS) was added to each well for 4 h. Next, the formazan crystals were dissolved in 100% DMSO, and absorbance was measured at 570 nm using Perkin Elmer EnSpire multimode plate reader. At least three independent experiments were performed. In the SRB assay, 100 μL of 10% (*w*/*v*) aqueous solution of the ice-cold trichloroacetic acid was added for 1 h. Plates were washed with water, allowed to air-dry, and stained with 100 μL of 0.4% sulforhodamine B solution in 1% acetic acid for 15 min. The cells were washed five times with 1% acetic acid (100 μL) and dried. After addition of 10 mM Tris base (pH 10.5, 150 μL/well), absorbance was measured at 570 nm using Perkin Elmer EnSpire multimode plate reader (PerkinElmer, Inc., Waltham, MA, USA). Data were obtained from at least five independent experiments. 

### 2.8. EB/AO Staining

The A549 cells with inducible expression of the *HtrA4* gene were seeded on coverslips placed in a 12-well plate, at a density of 5 × 10^5^ cells per well. To induce *HtrA4* expression, 1 μg/mL of doxycycline was added to the medium 24 h before treatment with etoposide. The cells were treated with 20 µM etoposide for 48 h. Control samples were treated with etoposide but not with doxycycline. The cells were stained with a mixture of acridine orange (4 µg/mL) and ethidium bromide (4 µg/mL) in PBS, for 5 min. The slides were washed with warm PBS, attached to a microscope slide, and examined under a fluorescent microscope (Zeiss Axio, Oberkochen, Germany).

### 2.9. Cell Death Analysis

The A549 cells (1 × 10^5^) with inducible *HtrA4* expression were seeded per well in 6 well-plates. To induce the *HtrA4* expression, 1 μg/mL of doxycycline was added to the medium 24 h before treatment with etoposide. The cells were treated with 15 µM etoposide for 48 h. Control cells (A549) were treated with etoposide and with/without doxycycline for 48 h, like the transduced cells. The trypsinized cells and medium were collected and centrifuged for 10 min at 300× *g*. The populations of cells (%) in the early (annexin-V+/7-AAD−) and late stages of apoptosis (annexin-V+/7-AAD+) were determined using the Muse Annexin V and Dead Cell Assay Kit and Muse™ Cell Analyzer (Millipore, Hayward, CA, USA).

### 2.10. Cell Cycle Analysis

The cells were prepared in the same way as described above in the cell death analysis. The cells were washed with ice-cold PBS, followed by processing with the Muse^TM^ Cell Cycle Kit according to the manufacturer’s instructions (http://www.icms.qmul.ac.uk/flowcytometry/uses/musekits/protocols/MCH100106%204600-3387MAN%20[B]%20MUSE%20CELL%20CYCLE%20KIT%20USER%27S%20GUIDE.pdf) and analyzed using the Muse™ Cell Analyzer (Millipore, Hayward, CA, USA).

### 2.11. Clonogenic Assay

A549 cells with inducible *HtrA4* expression were seeded at 800 cells/well in 6-well plates, cultured overnight, and treated with etoposide for 24 h. To induce the *HtrA4* expression, 1 μg/mL of doxycycline was added to the medium 24 h before treatment with etoposide. Control samples were treated with etoposide but without doxycycline. After 2 weeks, colonies were fixed with acetic acid/methanol 1:7 (*v*/*v*) and stained with 0.5% crystal violet. Colonies were counted manually.

### 2.12. Wound Healing Assay

The cells were seeded in 6-well plates (3 × 10^5^) and cultured to reach ~80% confluence. Then, the monolayer was scratched with a 200 μL pipette tip across the center of the well. The cells were washed with medium to remove the detached cells and photographed under a microscope at various time intervals (Zeiss Axio, Oberkochen, Germany). The gap distance was quantitatively evaluated using ImageJ software 1.52n. At least 10 different pictures were analyzed for each variant.

### 2.13. Statistical Analyses

Data are expressed as means ± SD of at least three independent experiments. Comparative data were analyzed with the unpaired or paired Student’s *t*-test using GraphPad Prism software 5.0. The results were considered statistically significant when the *p*-value was less than 0.05 (*), 0.01 (**), or 0.001 (***).

## 3. Results

### 3.1. HtrA4 Is a Secreted Protein but Also Co-Localizes with Mitochondria and Is Present in the Cytoplasm

**Figure 1:**
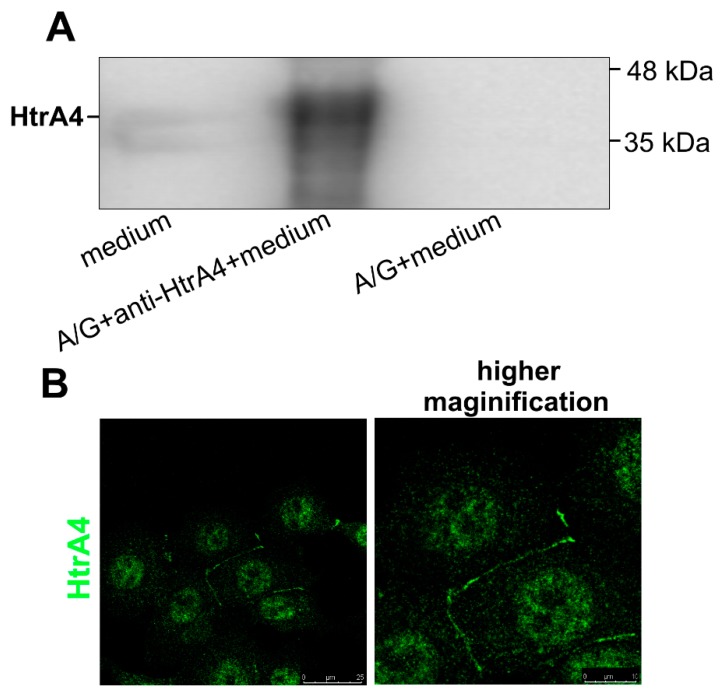
HtrA4 is a secreted protein. The medium of the MCF7 cultured cells containing endogenous HtrA4 was immunoprecipitated with the anti-HtrA4 antibodies and A/G agarose resin, analyzed by SDS-PAGE and subsequently probed with the anti-HtrA4 antibodies. The representative result of western blotting is shown in (**A**). In (**B**), the MCF7 cells are visualized by fluorescence microscopy. The HtrA4 protein was labeled using the anti-HtrA4 antibodies and secondary anti-rabbit antibodies conjugated with Alexa 488.

The HtrA4 protein possesses a similar signal secretory peptide as the HtrA1/3, and it has been previously shown to be exported to medium of cultured cells [18]. However, since the HtrA1/3 were found to function not only in cellular matrix but also to play important roles inside the cell [10,29,30,31], we checked cellular localization of HtrA4. We found that indeed, HtrA4 was secreted into the medium by the cultured MCF7 breast cancer cells, which contained a relatively high level of endogenous HtrA4 (Figure 1A and Figure S1). Moreover, using fluorescence microscopy, we observed a specific localization of HtrA4 in the places of contact between two cells, probably caused by blocking the HtrA4 secretion or by its trapping between the cells (Figure 1B). The simplest explanation of this phenomenon would be that at the places of tight contact, the HtrA4 secretion/diffusion into the medium is physically blocked.

**Figure 2:**
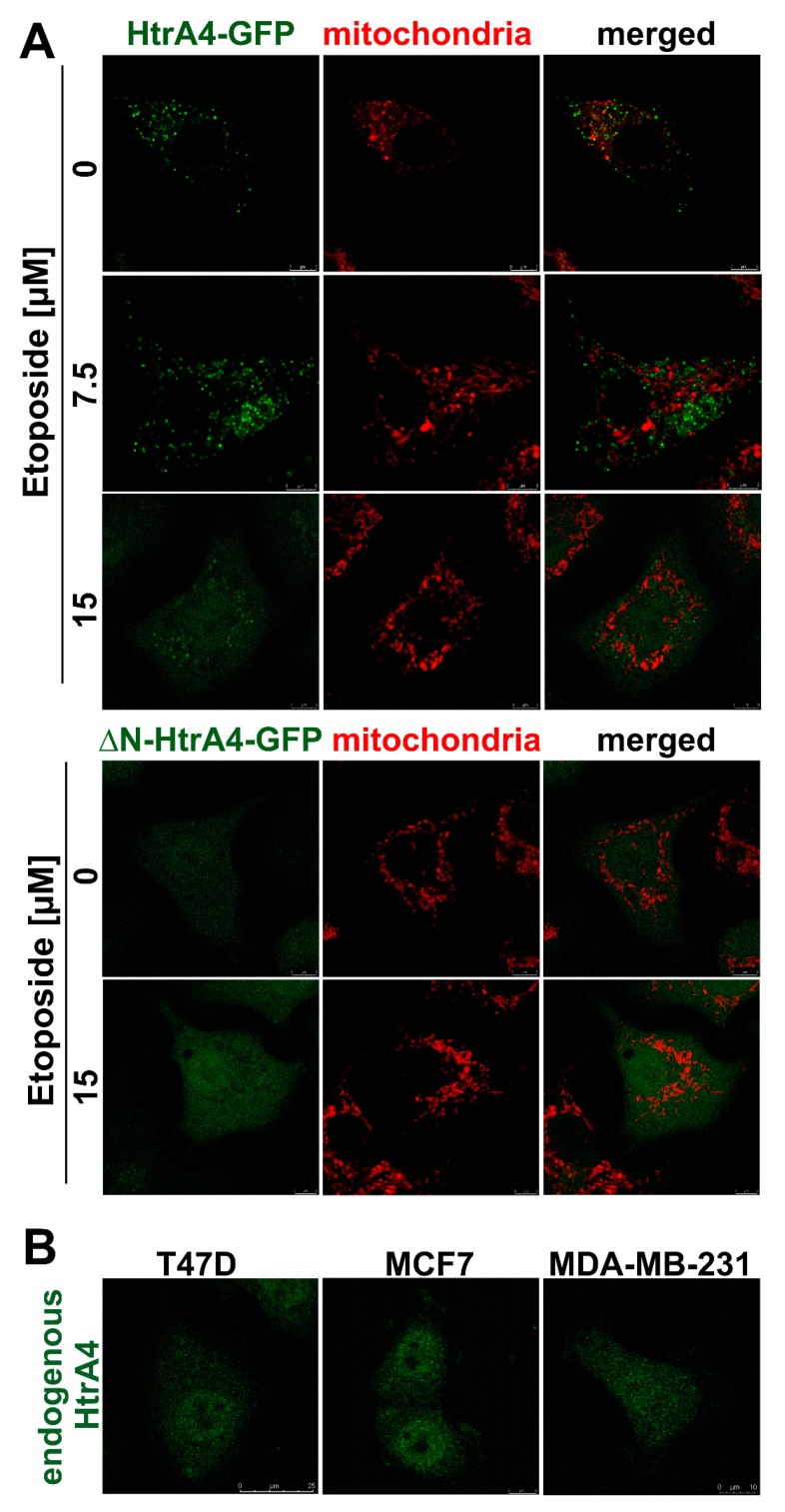
Localization of HtrA4 in the cell. The lung cancer (A549) cells with stable exogenous production of the HtrA4-GFP and ΔN-HtrA4-GFP fusion proteins (**A**), and the breast cancer T47D, MCF7, and MDA-MB-231 cells with endogenous HtrA4 (**B**) were analyzed by fluorescent microscopy. Endogenous HtrA4 was labeled with Alexa 488 (green) and mitochondria with MitoRED (red). The A549 cells were treated with etoposide as indicated in the panels.

However, we also demonstrated the intracellular localization of HtrA4. Since it has been previously shown that localization of the HtrA1-3 proteins depends on the presence or absence of their N-terminal domains [29,30,32,33], we used the lung cancer cells (A549) with exogenous expression of the full-length HtrA4 or of its N-terminally truncated variant, called ΔN-HtrA4, both tagged with the GFP protein as a fluorescent marker. It should be noted that the ΔN-HtrA4 variant contains the region required for trimerization [12] and forms trimers in solution [12,34]. We found that under normal conditions, the full-length HtrA4 formed clusters in cytoplasm and also partially colocalized with mitochondria (confirmed by statistical analysis) (Figure 2A, Table 1), while the ΔN-HtrA4 formed a diffused pattern, typical for cytoplasmic proteins, resembling the pattern observed before for HtrA1/3 [10,27,29,30]. We expect that the HtrA-containing punctate structures other than mitochondria are the ER and Golgi involved in the HtrA4 protein transport outside the cell. Under stressful conditions induced by etoposide, we observed the disappearance of the mitochondria-connected HtrA4 and appearance of the diffused, cytoplasmic pattern; the cytoplasmic HtrA4 clusters were transformed in a similar way (Figure 2A, Table 1). A similar cytoplasmic localization was found in the MCF7, MDA-MB231, and T47D breast cancer cells, which contained endogenous HtrA4 mainly in a truncated form (Figure 2B and Figure S1).

### 3.2. HtrA4 Decreases Survival of Cancer Cells

**Figure 3:**
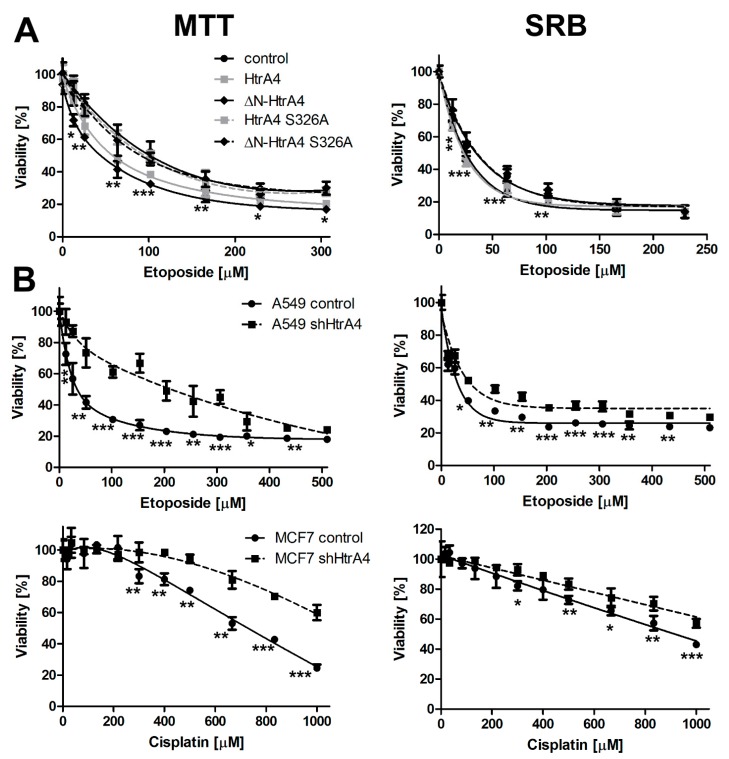
HtrA4 decreases the viability of cancer cells. The assay was performed using the A549 cells with exogenous HtrA4 and ΔN-HtrA4, induced by adding doxycycline to the medium (**A**) and the A549 and MCF7 cells with downregulated *HtrA4* expression (**B**). The survival is presented as a percent of live cells containing exogenous HtrA4 (induced by doxycycline) versus not containing HtrA4 (not induced by doxycycline). For more clarity, only the data of the A549 transduced with pTW_H4 noninduced control cells are presented (in **A**). Viability of cancer cells was analyzed using MTT and SRB assays. Data correspond to mean ± SD of at least five experiments. *p*-value < 0.05 (*), 0.01 (**), or 0.001 (***).

In the next step, we investigated whether HtrA4 influenced cell death. To achieve this goal, we constructed a set of the A549 cell lines with exogenous, doxycycline-induced expression of HtrA4, ΔN-HtrA4, and their proteolytically inactive variants (with the S326A substitution in the catalytic triad). A549 adenocarcinoma cells were used because they contained a relatively low level of endogenous HtrA4 (Figure S1). Additionally, these cells do not produce endogenous HtrA3, which has a significant effect on cell death [10,30] and whose cellular interacting partners are similar to those of HtrA4 [27,34]. However, A549 cells express the HtrA1 and 2 proteins [35,36]. The cells induced by doxycycline to produce HtrA4 were treated with increasing concentrations of etoposide and analyzed by the MTT (indicating succinate dehydrogenase activity) and SRB (indicating total protein level) methods. The results showed that the exogenous production of either HtrA4 or ΔN-HtrA4 significantly decreased survival of the drug-treated cancer cells compared to the control, noninduced cells. However, a stronger effect was caused by the truncated variant, ΔN-HtrA4 (Figure 3A). The cells producing the proteolytically inactive HtrA4 did not substantially affect cell viability (Figure 3A), which suggests that the process of cell death stimulation by HtrA4 is dependent on its proteolytic activity. A comparison of the levels of the overproduced HtrA4 proteins is presented in Figure S2C to exclude the possibility that the observed low impact of the inactive (S326A) forms might be due to their lower level.

To confirm that HtrA4 promotes cell death, we used a reverse, complementary approach and downregulated the HtrA4 expression by shRNA in the A549 lung cancer cells. Additionally, we modulated HtrA4 production in breast cancer cells (MCF7) that contained a high level of endogenous HtrA4 (Figure S1). The effect of HtrA4 downregulation on cell viability was monitored in the A549 cells exposed to etoposide and in the MCF7 cells treated with cisplatin. As shown by the MTT and SRB tests (Figure 3B), HtrA4 silencing significantly increased the viability of drug-treated lung and breast cancer cells in comparison to the control cells.

The levels of the HtrA4 proteins in the cells overproducing them and in cells with the silenced HtrA4 gene are shown in Figure S2.

Taken together, these results indicate that the full-length HtrA4 and its N-terminally deleted variant promote cancer cell death induced by chemotherapeutic drugs. The effect is dependent on the HtrA4 proteolytic activity, and the N-terminally deleted HtrA4 is more efficient in cell death promotion.

### 3.3. HtrA4 Promotes Cell Death by Enhancing Apoptosis

**Figure 4:**
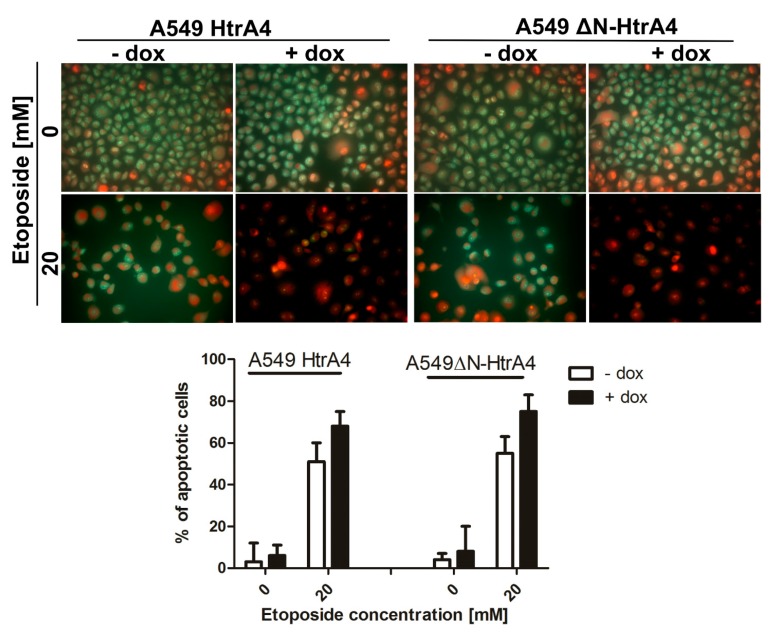
The influence of HtrA4 on viability of cancer cells assayed by AO/EB double staining. The analysis of live (green) and apoptotic cells with concentrated and asymmetrically localized orange-red nuclei was performed using fluorescence microscopy. The A549 cells with exogenous HtrA4 and ΔN-HtrA4 induced by doxycycline were tested.

**Figure 5:**
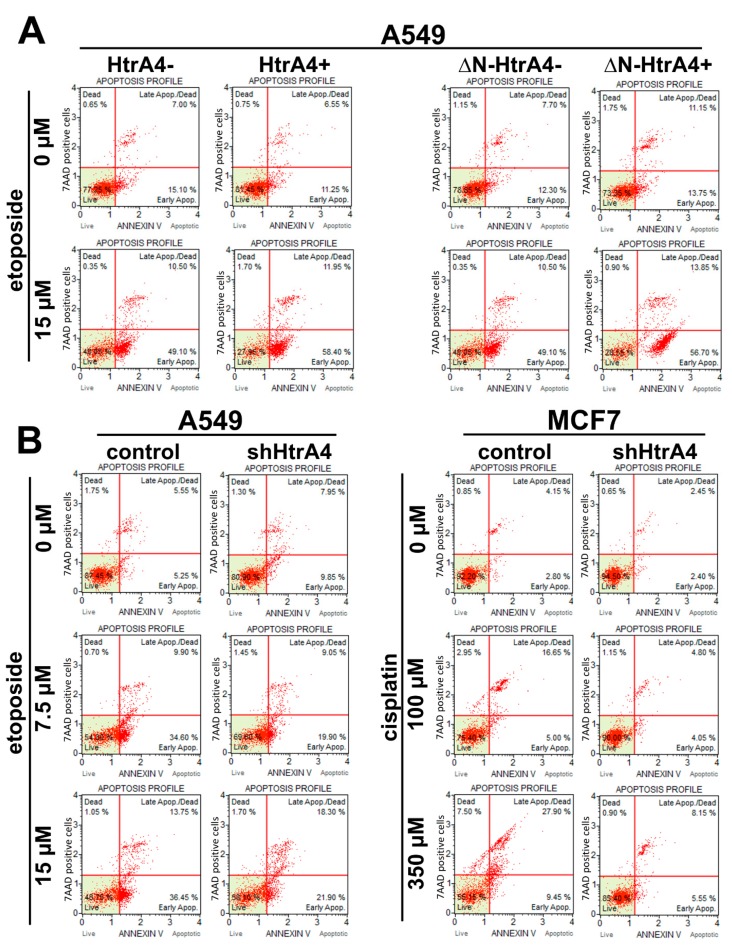
HtrA4 promotes cell death of cancer cells via induction of apoptosis. The A549 cells with exogenous HtrA4 or ΔN-HtrA4 induced by adding doxycycline to the medium (**A**) and the A549 and MCF7 cells with the silenced *HtrA4* expression (**B**) were analyzed using the Muse Annexin V and Dead Cell Assay Kit and Muse™ Cell Analyzer. Representative plots are shown. Quantitative data are presented in Table 2.

Subsequently, we determined the type of cell death induced by HtrA4. Using the AO/EB double staining and flow cytometry connected with annexin V and 7-aminoactinomycin D staining, we showed that exogenous production of the HtrA4/ΔN-HtrA4 in the lung adenocarcinoma A549 cells decreased the viability of etoposide-treated cancer cells by increasing the percentage of cells in the early and late stages of apoptosis (Figure 4 and Figure 5A, Table 2). A reversed effect was observed in the cells with downregulated HtrA4 expression—these cells had a higher percentage of live cells and lower of cells in the early and late stages of apoptosis compared to the controls (Figure 5B, Table 2). In conclusion, these results showed that HtrA4 promotes death of the chemotherapeutic drug-treated cancer cells by enhancing apoptosis.

### 3.4. HtrA4 Enhances the Effect of Chemotherapeutic Agents on Clonogenic Potential and Motility of Cancer Cells

**Figure 6:**
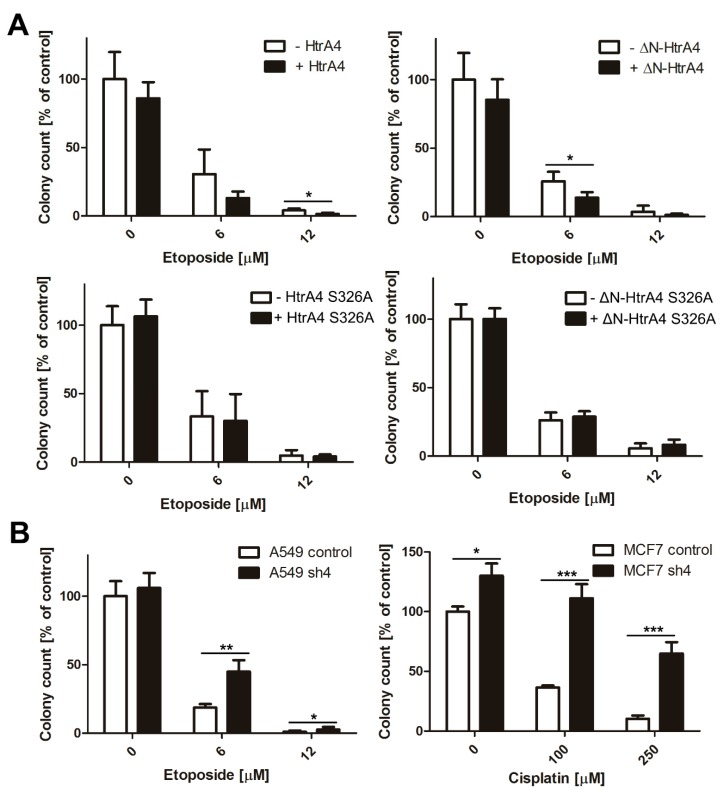
HtrA4 reduces the clonogenic potential of cancer cells. The A549 cells with inducible expression of the *HtrA4* or *ΔN-HtrA4* genes (**A**) and the A549 and MCF7 cells with downregulated *HtrA4* expression (**B**) were treated with a chemotherapeutic for 24 h, and the clonogenic potential was assayed. The colonies were stained by crystal violet (Figure S3), and quantitative results are shown as mean ± SD of three independent experiments. *p*-value < 0.05 (*), 0.01 (**), or 0.001 (***).

**Figure 7:**
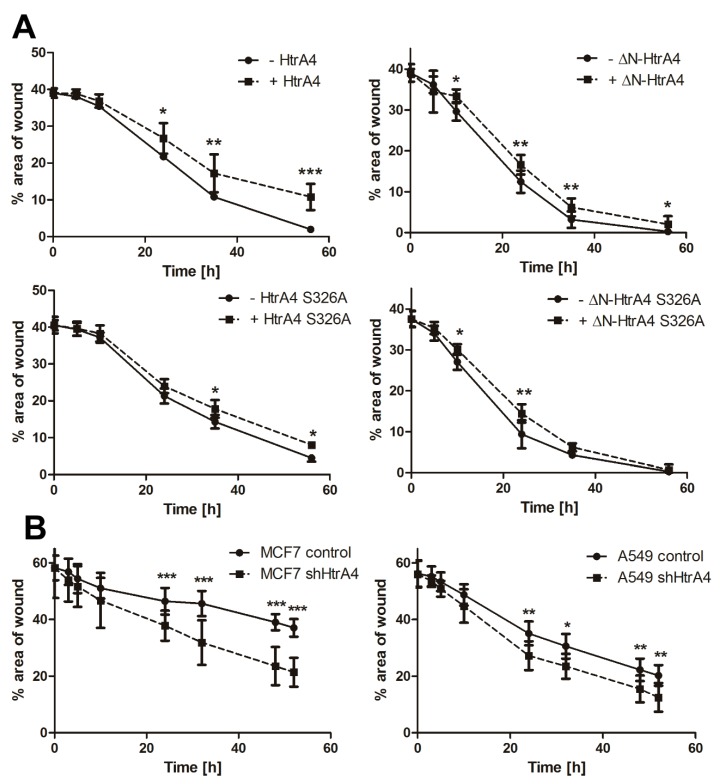
HtrA4 reduces the motility of cancer cells. The lung cancer A549 cells with exogenous production of HtrA4, ΔN-HtrA4, or their inactive variants had a decreased ability to migrate (**A**), while the MCF7 and A549 cells with the *HtrA4* gene expression silenced by shRNA migrated more efficiently (**B**) compared to the control cells. The analysis was performed using a wound healing assay in two independent approaches with at least ten photos. Representative images are shown in Figure S4. The data are presented as means ± SD. *p*-value < 0.05 (*), 0.01 (**), or 0.001 (***).

Since the HtrA4 protein affected cell survival, we investigated its influence on clonogenic potential and motility of cancer cells treated with chemotherapeutics. We found that exogenous production of the HtrA4 and ΔN-HtrA4 proteins significantly reduced clonogenic potential (Figure 6A) as well as ability to migrate (Figure 7A) of etoposide-exposed lung cancer cells in comparison to controls. This effect was weaker or not observed in the cells with proteolytically inactive HtrA4 proteins, which confirms the importance of the HtrA4 proteolytic activity in the induction of cell death mechanisms (Figure 6A and Figure 7A).

**Figure 8:**
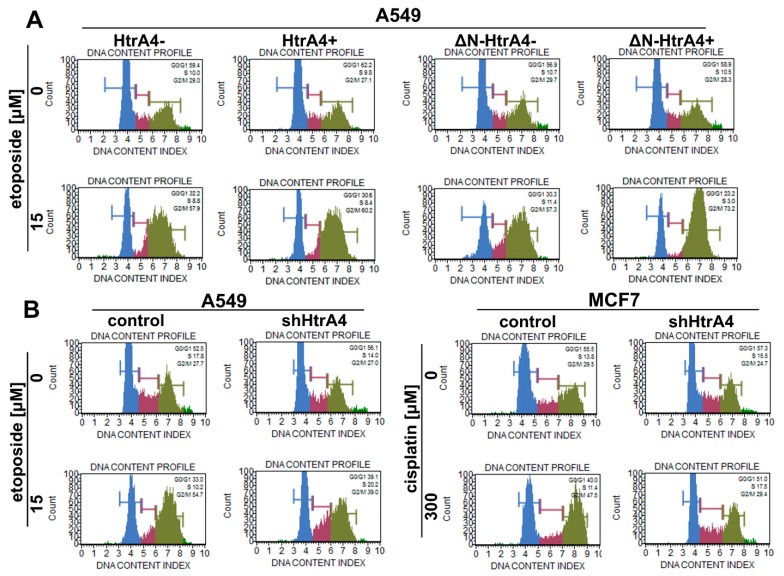
HtrA4 promotes the influence of chemotherapeutic drugs by increasing the G2/M arrest of cancer cells. The A549 cells with exogenous HtrA4 or ΔN-HtrA4 induced by adding doxycycline to the medium (**A**) and the A549 and MCF7 cells with suppression of the *HtrA4* gene (**B**), treated with an appropriate chemotherapeutic (as described in the panel), were analyzed using the Muse Cell Cycle Kit and Muse™ Cell Analyzer. Representative plots are shown. The results are quantitatively presented in Table 3.

Moreover, because of the interaction of HtrA4 with cytoskeleton proteins [34], we also investigated its influence on cell cycle. As can be seen in Figure 8A and Table 3, exogenous production of either HtrA4 or ΔN-HtrA4 proteins in lung cancer cells treated with etoposide increased arrest of the cells in the G2/M phase, thus enhancing the effect of the chemotherapeutic agent.

To confirm these conclusions, we used the lung and breast cancer cells with suppression of the *HtrA4* gene by shRNA. The modulated cells with silenced *HtrA4* gene, exposed to a chemotherapeutic, had a significantly higher clonogenic potential (Figure 6B) and motility compared to the control cells (Figure 7B). Moreover, we noted that these cells, when drug-exposed, exhibited substantially less blocking at the G2/M phase compared to the nonsilenced controls (Figure 8B, Table 3).

Summing up, these results indicate that HtrA4 is an important factor that enhances the influence of drugs routinely used in chemotherapy on clonogenic potential and motility of cancer cells, and it increases blocking of cell cycle at the G2/M phase.

**Figure 9:**
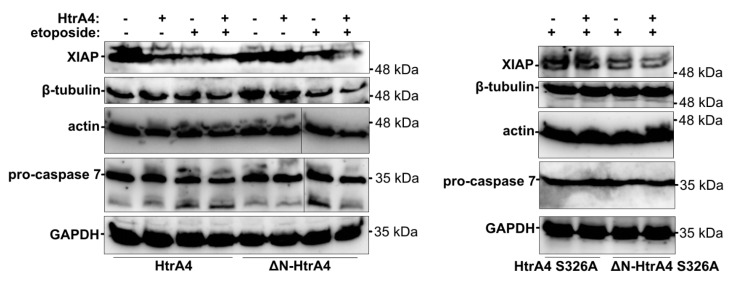
HtrA4 promotes degradation of XIAP and, less efficiently, of β-tubulin, actin, and pro-caspase 7. The A549 cells with exogenous HtrA4, ΔN-HtrA4, or their inactive variants (+) induced by adding doxycycline to the medium were treated with 15 µM etoposide for 48 h. Control cells (−) were incubated with etoposide but without doxycycline. The cells and medium were collected and probed with specific antibodies. Representative blots are presented. Densitometric analyses of the immunoblotting results are shown in Figure S5.

To gain insight into the mechanism of HtrA4 action, we investigated the influence of exogenous production of HtrA4 on the level of selected cellular proteins. Using western blotting we showed that, under apoptotic conditions induced by etoposide, the HtrA4 and ΔN-HtrA4-producing cells had a reduced level of XIAP and also of β-tubulin, actin, and pro-caspase 7 compared to the control cells without exogenous HtrA4 (Figure 9 and Figure S5). This effect was not observed in the cells producing the proteolytically inactive HtrA4 and ΔN-HtrA4 variants (Figure 9, Figures S5 and S6A), which suggests that HtrA4 may promote apoptosis by degrading the anti-apoptotic XIAP protein and also by proteolysis of cytoskeletal structural proteins (actin and β-tubulin). Degradation of the executioner pro-caspase 7 may have a modulatory effect on apoptosis.

Additionally, under apoptotic conditions, the XIAP level in the cells with silenced *HtrA4* gene decreased to a lesser extent compared to the cells with functional HtrA4 (Figure S6B). This further supports the correlation between the degradation of XIAP by HtrA4 and cell death.

## 4. Discussion

HtrA4 is one of the four HtrA serine proteases that are involved in keeping order in the cell. It was previously shown that HtrA4 plays a crucial role in the implantation of the embryo, and its increased level is connected to pre-eclampsia [16,17,18,19,20]. Moreover, the changed levels of *HtrA4* expression were observed in brain tumors and the breast and prostate cancers in comparison to the normal tissues, which suggests HtrA4 involvement in oncogenesis [21]. So far, the knowledge regarding the role of HtrA4 in physiological and pathological processes is very limited. The high similarity of the HtrA4 protein domain structure to that of HtrA1 and HtrA3 and implication of HtrA4 in oncogenesis suggested that it may function similarly to the HtrA1/3, which act as tumor suppressors and promote cell death [10,27,29,30,31]. HtrA2 is also involved in oncogenesis and acts as a pro-apoptotic factor under conditions of stress (reviewed in [3,21]).

HtrA4 possess a similar signal secretory peptide as HtrA1/3, which are classified as secreted proteins and are present in the extracellular matrix [2,4]. It was shown previously that HtrA4 is secreted into maternal circulation [17]. The secreted fraction of HtrA4 was also demonstrated in the medium of BeWo cultured cells [18]. Here, we showed that HtrA4 is secreted into the medium by the MCF7 cells, thus confirming the secretory nature of HtrA4 (Figure 1).

Moreover, we investigated the intracellular localization of the HtrA4. We found that, under normal conditions, the full-length exogenous HtrA4 partially co-localized with mitochondria (Figure 2A, Table 1), while during apoptotic stress induced by etoposide, a typical diffused, cytoplasmic pattern was observed. Interestingly, the N-terminally deleted ΔN-HtrA4 showed cytoplasmic patterns also in the drug’s absence (Figure 2A, Table 1). A similar cytoplasmic localization was found in the MCF7, MDA-MB231, and T47D breast cancer cells, which contain endogenous, truncated HtrA4 (Figure 2B and Figure S1). These results collectively suggest that, upon stress, the HtrA4 N-terminal domain may be cleaved, and the truncated protease migrates to cytoplasm. A similar phenomenon was observed in the case of the HtrA1/3 proteases—their processed forms (i.e., without the N-terminal domain) have been found in the cytoplasm where they perform important physiological functions [29,30,32]. In response to cytotoxic stress induced by chemotherapeutic agents, the HtrA1/3 expression was upregulated, and the HtrA1/3 proteins underwent activation correlated with limited proteolysis, resulting in the removal of the N-terminal Mac25 domain and yielding a 35 kDa product [29,30]. Moreover, it was shown that a fraction of full-length HtrA3, despite the lack of mitochondrial targeting sequence, was located in mitochondria, and in cancer cell lines treated with chemotherapeutic HtrA3, it was translocated from the mitochondria to the cytosol [30]. A similar event was observed in the case of HtrA2. Following apoptotic stimuli, HtrA2 is released from the mitochondria to the cytosol where it degrades the inhibitor of apoptosis proteins (IAPs), including XIAP, and thus contributes to the induction of apoptosis. The release from mitochondria is accompanied by the autoproteolytic cleavage of the HtrA2 N-terminal domain, which increases HtrA2 proteolytic activity and creates a new N-terminus with the AVPS (Ala-Val-Pro-Ser) motif, important for binding of the IAPs, including XIAP [33,37].

Subsequently, in this work, we for the first time showed that HtrA4 reduces survival of adenocarcinoma cells treated with chemotherapeutic drugs (Figure 3A,B) and promotes cancer cell death by enhancing apoptosis (Figure 4 and Figure 5A, Table 2). This process was dependent on the proteolytic activity of HtrA4, as previously observed for in the HtrA1 and HtrA3 [10,30,31]. Since a stronger pro-apoptotic effect was caused by the abbreviated HtrA4 variant (ΔN-HtrA4) (Figure 3A), it is possible that removal of the N-terminal domain leads to increase of the HtrA4 proteolytic activity. Such activity increase is consistent with the earlier studies on the HtrA1-3 proteases [30,31,33].

Moreover, we demonstrated that exogenous production of HtrA4 and ∆N-HtrA4 attenuated the clonogenic potential (Figure 6A) and motility (Figure 7A) of cancer cells treated with etoposide, and it modulated the cell cycle by enhancing arrest of the cells in the G2/M phase (Figure 8A, Table 3). These results were confirmed by showing that downregulation of HtrA4 in the lung and breast cancer cells contributed to increased survival (Figure 3B and Figure 5B, Table 2), clonogenic potential (Figure 6B), and motility of cancer cells (Figure 7B). A decreased blocking of the cells in the G2/M phase was also observed (Figure 8B, Table 3). Collectively, these results show that HtrA4 enhances the activity of chemotherapeutics by decreasing survival of cancer cells as well as their clonogenic potential and motility, and the latter are tightly connected with metastasis.

It may be noted that the HtrA4 knockdown was more effective on cell death, cell cycle arrest, clonogenic potential, and cell motility than the HtrA4 overexpression (Figure 3, Figure 5, Figure 6, Figure 7, Figure 8, Table 2 and Table 3). It is possible that the level of endogenous HtrA4 may be sufficient to maintain its anti-survival function in the cell; thus, increasing its pool has a moderate effect. Also, the additional, overproduced HtrA4 may be disabled by cellular inhibitor(s) whose presence has been suggested previously [34]. On the other hand, the HtrA4 function may be important, and the lack of this protein causes a definite increase in pro-survival cellular activities.

Our finding that HtrA4 can be an important factor that regulates the motility of cells is in agreement with the study of Liu et al. (2018), who showed that ectopic expression of HtrA4 significantly decreased trophoblast cell migration [20]. The involvement of HtrA4 in the regulation of cell cycle genes in umbilical vein endothelial cells was also observed [26].

Our earlier results showed that HtrA4 formed complexes in vitro and *in cellulo* with structural cytoskeleton proteins (β-tubulin and actin), anti-apoptotic protein (XIAP), and the executioner pro-caspase 7 and degraded these proteins in vitro [34]. Here, we found that under stressful conditions induced by the etoposide treatment, HtrA4 and ΔN-HtrA4 decreased the level of cellular XIAP and, less efficiently, of β-tubulin, actin, and pro-caspase 7 (Figure 9 and Figure S5). These results suggest that HtrA4 may promote cell death by degradation of the anti-apoptotic XIAP and of cytoskeletal structural proteins (actin and β-tubulin); it may also affect cell death by degrading pro-caspase 7. Interestingly, the overexpression of the full-length HtrA4 without induction of apoptosis induced degradation of XIAP (Figure 9, left panel) but did not induce cell death (Figure 4). This suggests that XIAP degradation by itself may not be sufficient to cause cell death. It is known that initiation of apoptosis is connected with imbalance between multiple pro- and anti-apoptotic signals [38]. Thus, it is possible that the degradation of XIAP by HtrA4, in order to promote apoptosis, needs other pro-apoptotic events to occur in parallel, especially in the cancer cells that usually have mutations inhibiting apoptosis.

It has been shown that HtrA1-mediated apoptosis is associated with degradation of XIAP and caspases 3 and 7 [7,31], while the HtrA3 and HtrA2 proteases promote apoptosis mainly by XIAP hydrolysis [10,37]. Additionally, it was shown that HtrA1 and 3 modulate microtubule stability, cytoskeleton dynamics, and cell motility [27,29,39,40]. Thus, the mechanisms of the human HtrA proteases action in cell death promotion seem to be similar. It is possible that the HtrAs may have a cumulative effect, or function independently, since the level of HtrA proteins is tissue-specific and altered in many diseases [3,4]. Involvement of several HtrA proteases in cell death pathways creates a system whose regulation may be finely adjusted to the actual stress conditions.

Summing up, our results provide new insight into the mechanism of the human HtrA4 protease action in cell death and oncogenesis. Since HtrA4 stimulates drug-induced death of cancer cells, this protease seems to be a promising therapeutic target. We believe that the results of this work may help to develop new anti-cancer therapeutic strategies.

## Figures and Tables

**Table 1:** Analysis of the HtrA4-GFP and mitochondria colocalization parameters in A549 cells. Pearson’s correlation and overlap coefficient were assigned by the LAS AF 3.3.0 software. The data correspond to mean ± SD of at least seven independent pictures.

HtrA4 Variant	Etoposide [µM]	Pearson’s Correlation	Overlap Coefficient
**HtrA4-GFP**	**0**	0.52 ± 0.09	0.58 ± 0.08
	**7.5**	0.17 ± 0.04	0.26 ± 0.03
	**15**	0.13 ± 0.03	0.23 ± 0.03
**∆N-HtrA4-GFP**	**0**	0.12 ± 0.02	0.23 ± 0.03
	**15**	0.11 ± 0.02	0.19 ± 0.03

**Table 2:** Viability analysis of cancer cells treated with a chemotherapeutic. The assay was performed using the Muse Annexin V and Dead Cell Assay Kit. The data were collected from at least three independent experiments. The representative assay results are graphically presented in Figure 5. *p*-value < 0.05 (*), 0.01 (**), 0.001 (***), or not significant (ns).

Cell Line	Chemotherapeutic Drug [µM]		HtrA4 Variant	Live Cells [%±SD]	Early Apoptotic Cells [%±SD]	Late Apoptotic/Dead Cells [%±SD]	Total Apoptotic Cells	
							[%]	Statistical Analysis
A549	Etoposide	0	− HtrA4	80.6 ± 4.7	12.9 ± 3.1	6.4 ± 0.8	19.3	ns
			+ HtrA4	80.9 ± 0.8	13.4 ± 3.0	5.3 ± 1.1	18.7	
		15	− HtrA4	39.0 ± 1.5	46.5 ± 3.7	12.7 ± 0.3	59.2	*
			+ HtrA4	29.4 ± 2.1	55.4 ± 4.3	14.4 ± 3.4	69.7	
		0	− ∆N-HtrA4	80.5 ± 2.3	10.7 ± 2.3	8.4 ± 0.9	19	ns
			+ ∆N-HtrA4	77.0 ± 5.1	12.2 ± 0.7	10.1 ± 1.4	22.4	
		15	− ∆N-HtrA4	37.9 ± 2.8	47.8 ± 3.5	13.0 ± 1.5	60.8	**
			+ ∆N-HtrA4	26.7 ± 2.6	57.5 ± 1.1	15.3 ± 2.1	72.8	
A549	Etoposide	0	control	86.8 ± 0.9	5.9 ± 0.9	6.7 ± 1.2	12.6	ns
			shHtrA4	85.1 ± 5.8	8.2 ± 2.3	6.4 ± 1.1	14.7	
		7.5	control	61.6 ± 7.1	26.65 ± 3.0	10.7 ± 0.5	37.3	ns
			shHtrA4	67.9 ± 3.8	19.0 ± 0.1	11.4 ± 2.0	30.4	
		15	control	51.8 ± 4.3	35.5 ± 1.3	11.8 ± 2.1	47.4	**
			shHtrA4	63.9 ± 3.1	20.9 ± 1.4	13.7 ± 2.0	34.6	
MCF7	Cisplatin	0	control	89.2 ± 4.2	3.3 ± 0.7	6.8 ± 0.2	10.1	ns
			shHtrA4	92.7 ± 2.5	2.8 ± 0.5	4.2 ± 0.4	7	
		100	control	73.9 ± 2.1	4.8 ± 0.2	18.0 ± 1.8	22.8	***
			shHtrA4	88.4 ± 2.3	4.0 ± 0.1	6.4 ± 0.5	10.3	
		350	control	46.8 ± 5.4	16.8 ± 2.6	30.9 ± 3.2	47.7	**
			shHtrA4	80.3 ± 7.3	6.3 ± 0.8	12.1 ± 1.5	18.4	

**Table 3:** Quantitative analysis of the percentage of cells in the cell cycle phases. This assay was performed using Muse Cell Cycle Kit. The data were obtained from at least three independent experiments. The representative assay results are graphically presented in Figure 8. *p*-value < 0.05 (*), 0.01 (**), 0.001 (***), or not significant (ns).

Cell Line	Chemotherapeutic Drug [µM]		HtrA4 Variant	Cell Cycle Phase					
				G0/G1		S		G2/M	
				[% ± SD]	Statistical Analysis	[% ± SD]	Statistical Analysis	[% ± SD]	Statistical Analysis
**A549**	Etoposide	0	− HtrA4	63.6 ± 3.8	ns	9.3 ± 1.2	ns	26.9 ± 2.2	ns
			+ HtrA4	64.1 ± 1.9		11.0 ± 1.9		24.7 ± 2.7	
		15	− HtrA4	32.5 ± 3.6	ns	10.0 ± 1.5	ns	53.3 ± 5.4	*
			+ HtrA4	29.6 ± 6.0		10.6 ± 1.9		58.6 ± 6.5	
**A549**	Etoposide	0	− ∆N-HtrA4	55.6 ± 2.1	ns	11.1 ± 2.7	ns	31.0 ± 1.4	ns
			+ ∆N-HtrA4	58.1 ± 2.9		11.5 ± 0.9		31.4 ± 2.5	
		15	− ∆N-HtrA4	29.2 ± 1.8	**	14.2 ± 2.3	**	54.5 ± 2.9	**
			+ ∆N-HtrA4	21.5 ± 2.4		4.4 ± 2.5		73.7 ± 5.1	
**A549**	Etoposide	0	control	56.1 ± 3.4	ns	15.8 ± 1.5	*	27.3 ± 1.4	ns
			shHtrA4	57.2 ± 1.2		13.6 ± 0.3		27.9 ± 1.0	
		15	control	32.4 ± 1.6	***	12.8 ± 1.9	*	55.7 ± 1.7	***
			shHtrA4	41.6 ± 2.4		16.2 ± 2.3		35.8 ± 2.0	
**MCF7**	Cisplatin	0	control	56.8 ± 3.3	ns	15.5 ± 1.9	ns	31.4 ± 2.9	ns
			shHtrA4	55.2 ± 4.1		17.9 ± 2.5		26.5 ± 3.2	
		300	control	41.5 ± 1.1	***	7.2 ± 2.5	**	48.5 ± 1.3	***
			shHtrA4	56.5 ± 3.5		13.4 ± 2.4		28.7 ± 1.2	

## Supplementary Materials

The following are available online at https://www.mdpi.com/2073-4409/8/10/1112/s1, Figure S1: Level of the endogenous HtrA4 protein in cell lines, Figure S2: The HtrA4 gene of the MCF7 and A549 cells was silenced by shRNA (A). The HtrA4 and ΔN-HtrA4 proteins were induced by doxycycline in the cells transduced with the appropriate plasmids (B). Comparison of the levels of the HtrA4 wt and S326A inactive proteins (C), Figure S3: HtrA4 reduces the clonogenic potential of cancer cells, Figure S4: HtrA4 decreases motility of cancer cells, Figure S5: The levels of the tested proteins in A549 cells exogenously producing HtrA4, ΔN-HtrA4 and their proteolytically inactive variants in normal and apoptotic (induced by etoposide treatment) conditions, Figure S6: The XIAP levels in the A549 cells with exogenous expression of HtrA4/ΔN-HtrA4 S326A (proteolytically inactive variants) (A) and A549 cells with the HtrA4 gene silenced by shRNA, under standard and apoptotic conditions induced by etoposide (B). Table S1: The plasmids used in this study.

## Abbreviations

HtrA	High-temperature requirement A
PD	protease domain
PDZ domain	Postsynaptic density protein 95, *Drosophila* disc large tumor suppressor and Zonula occludens-1 protein domain
XIAP	X-linked inhibitor of apoptosis protein
