# Supplementary material for: HtrA4 Protease Promotes Chemotherapeutic-Dependent Cancer Cell Death
Source: Cells. 2019 Sep 20;8(10):1112. doi: 10.3390/cells8101112 (PMC6829446; doi:10.3390/cells8101112)
Supplement: Supplementary file 1 [file cells-08-01112-s001.pdf]

HtrA4 Protease Promotes Chemotherapeutic-Dependent Cancer Cell Death

Tomasz Wenta [1,*], Michal Rychlowski [2], Miroslaw Jarzab [1] and Barbara Lipinska [1]

[1] Department of General and Medical Biochemistry, Faculty of Biology, University of Gdansk, Wita Stwosza 59, 80-308 Gdansk, Poland; miroslaw.jarzab@biol.ug.edu.pl (M.J.); barbara.lipinska@biol.ug.edu.pl (B.L.)

[2] Laboratory of Virus Molecular Biology, Intercollegiate Faculty of Biotechnology, University of Gdansk andMedical University of Gdansk, Abrahama 58, 80-307 Gdansk, Poland; michal.rychlowski@biotech.ug.edu.pl

* Correspondence: tomasz.wenta@biol.ug.edu.pl; Tel.: +48-58-5236055; Fax: +48-58-5236186

Table 1. The plasmids used in this study.

Plasmids	Relevant Characteristics	Reference or Source
pEGFP N1	Vector for fusing *EGFP* to the C-terminus of a partner protein	Clontech Inc.
pBabe puro	Retroviral expression vector	Cell Biolabs, Inc.
pCMV-VSV-G	Retroviral envelope vector	Cell Biolabs, Inc.
pRetroX-Tet-On Advanced	Retroviral vector for expressing rtTA-Advanced (reverse tetracycline-controlled transactivator protein rtTA)	Clontech, Inc.
pTW_H4	pRetroX-Tight pur *HtrA4* (amino acids 1-476)	this work
pTW_H4Q	pRetroX-Tight pur *HtrA4 S326A* (amino acids 1-476)	this work
pTW_ΔH4	pRetroX-Tight pur ΔN-*HtrA4* (amino acids 147-476)	this work
pTW_ΔH4Q	pRetroX-Tight pur *ΔN-HtrA4 S326A* (amino acids 147-476)	this work
pTW_H4-GFP	pBabe puro *HtrA4* (amino acids 1-476)	this work
pTW_ ΔH4-GFP	pBabe puro *ΔN-HtrA4* (amino acids 147-476)	this work
pMKO.1 puro	Retroviral vector for shRNA expression	Addgene; plasmid #8452
pTW_shHtrA4	pMKO.1 puro AAGCTACATACCCAGCCCTC – shRNA for *HtrA4*	this work

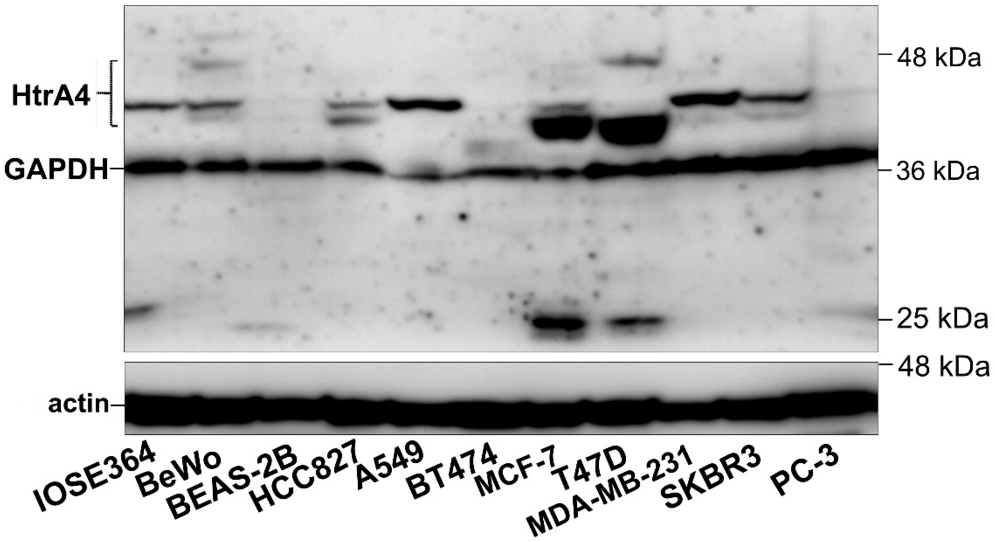

Figure 1. Level of the endogenous HtrA4 protein in cell lines. β-actin and GAPDH were used as the loading control.

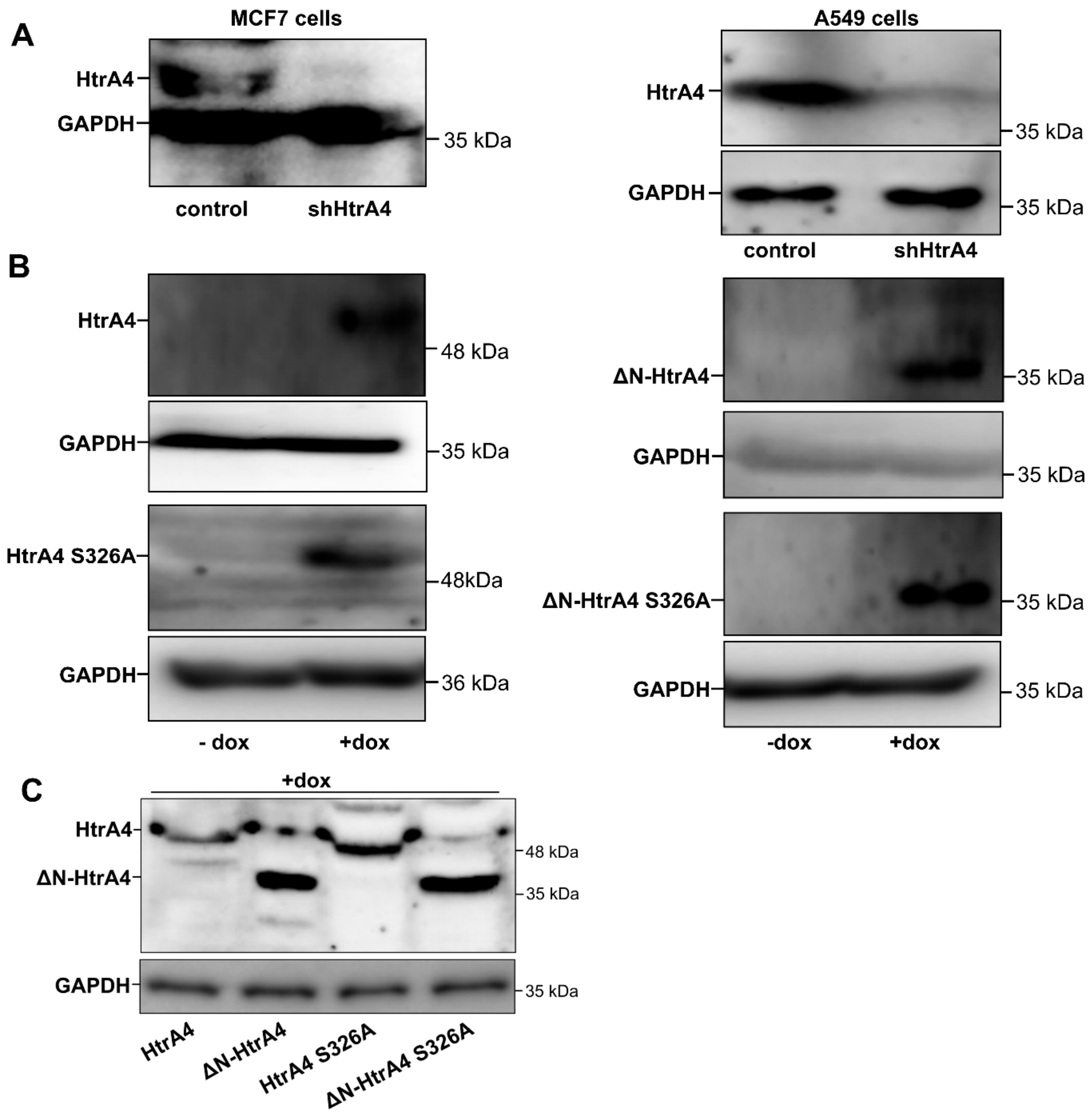

Figure S2. The HtrA4 gene of the MCF7 and A549 cells was silenced by shRNA (**A**). The HtrA4 and ΔN-HtrA4 proteins were induced by doxycycline in the cells transduced with the appropriate plasmids (**B**). Comparison of the levels of the HtrA4 wt and S326A inactive proteins (**C**). The immunoblotting was performed with the specific anti-HtrA4 and anti-GAPDH (loading control) antibodies.

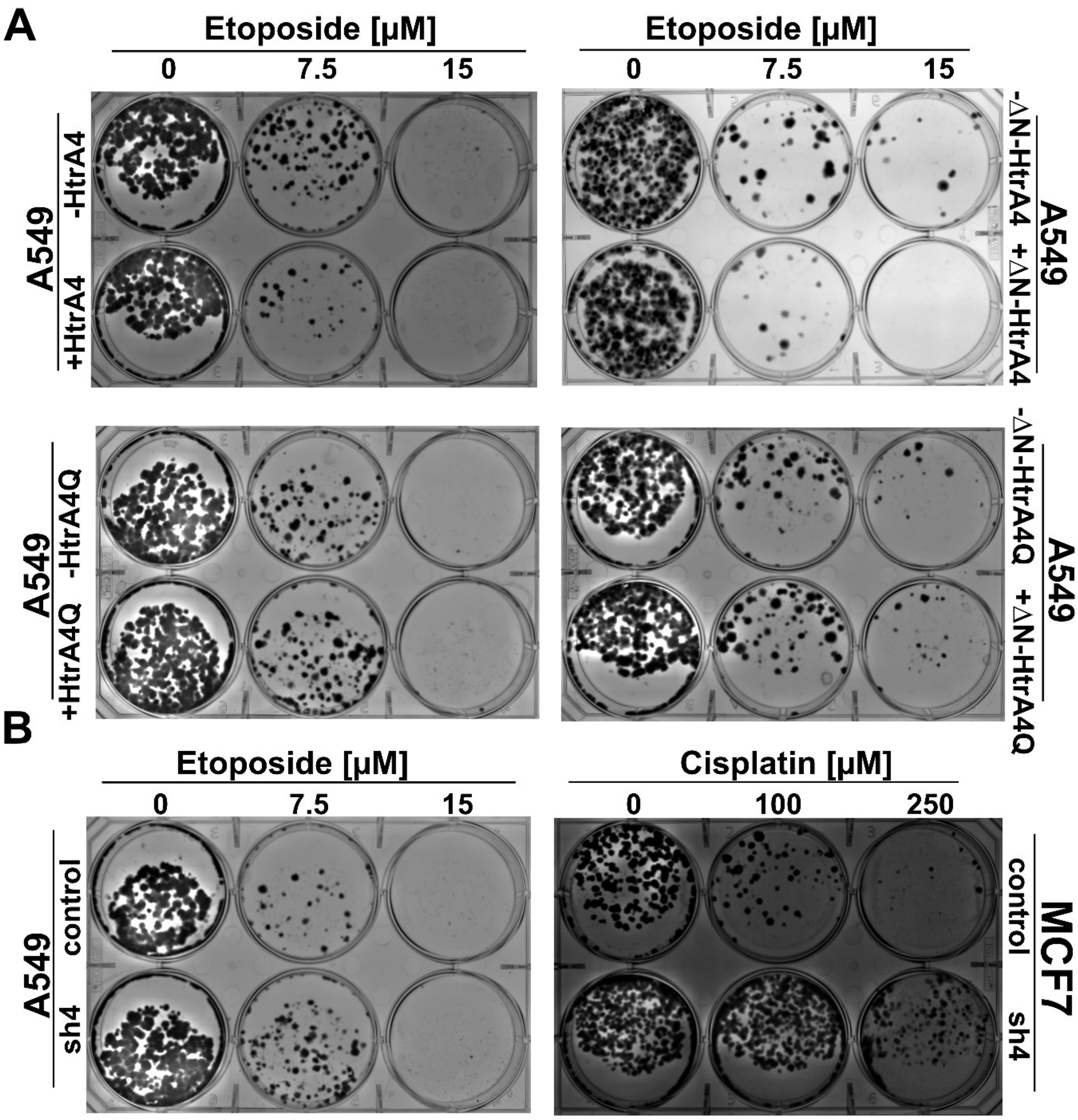

Figure S3. HtrA4 reduces the clonogenic potential of cancer cells. The colonies were stained with crystal violet and counted. Representative plate images are presented. The quantitative results are shown in Figure 6.

Time [h]	HtrA4-	HtrA4+	ΔN-HtrA4-	ΔN-HtrA4+
0	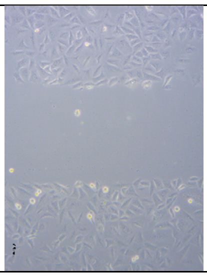	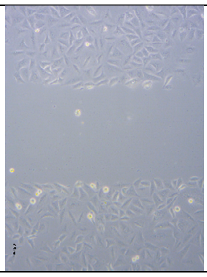	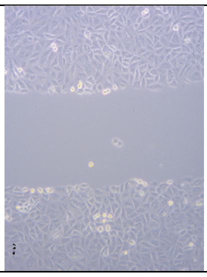	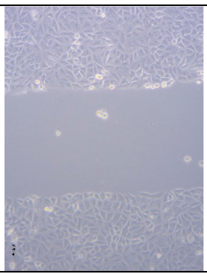

**5**	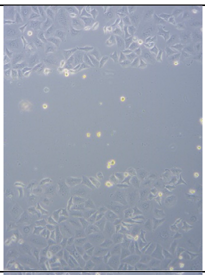	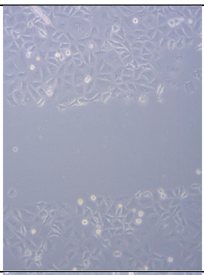	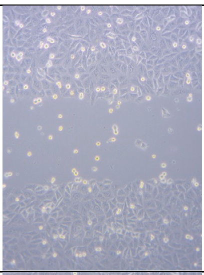	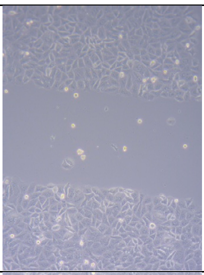
**10**	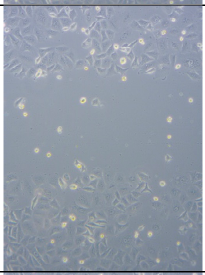	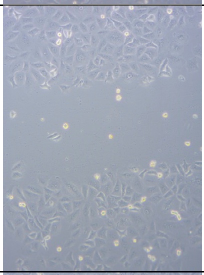	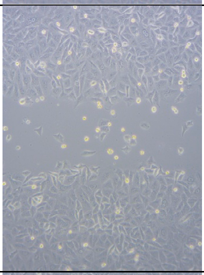	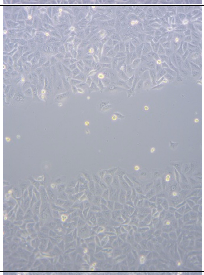
**24**	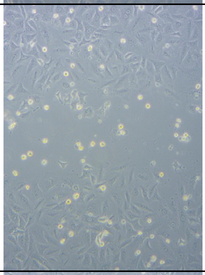	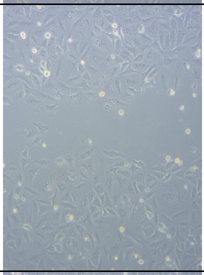	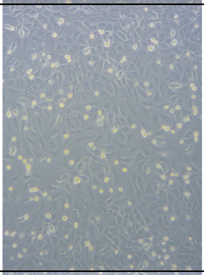	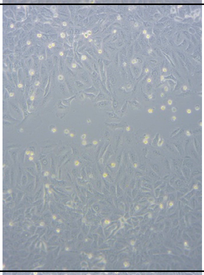
**35**	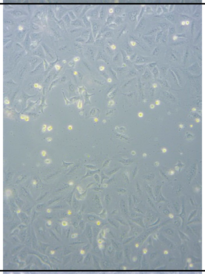	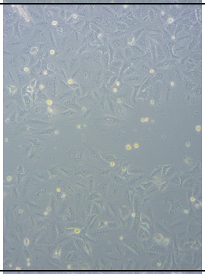	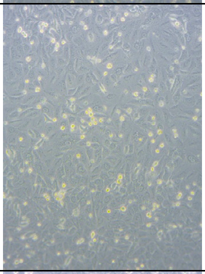	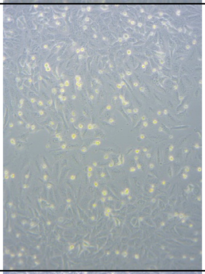
**56**	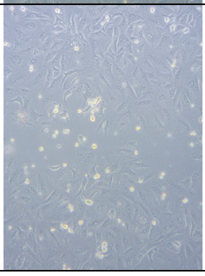	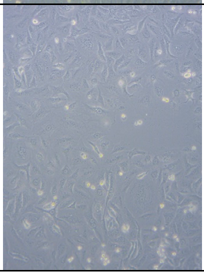	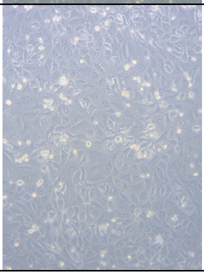	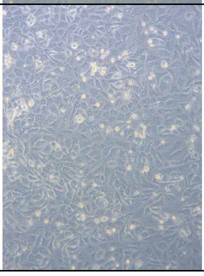

**Time [h]**	**- HtrA4 S326A**	**+ HtrA4 S326A**	**- ΔN-HtrA4 S326A**	**+ ΔN-HtrA4 S326A**
**0**	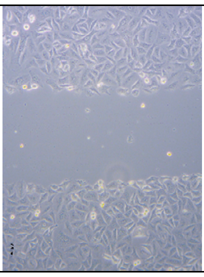	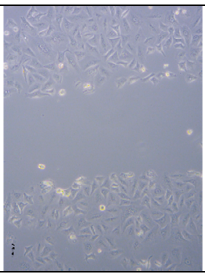	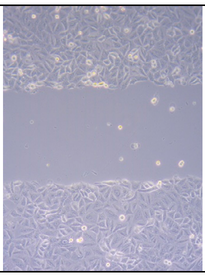	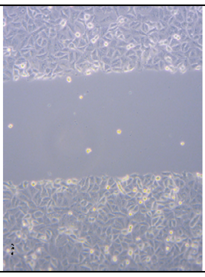

**5**	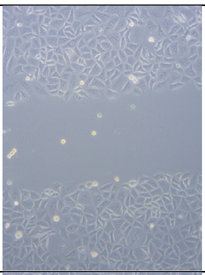	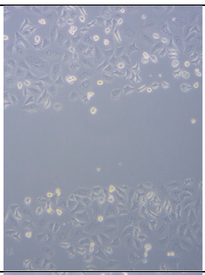	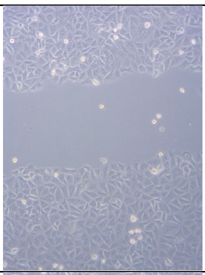	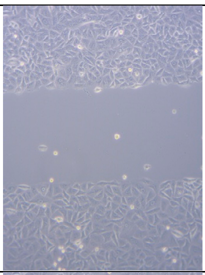
**10**	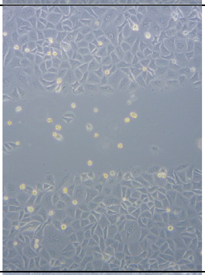	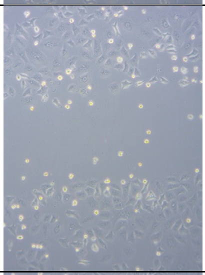	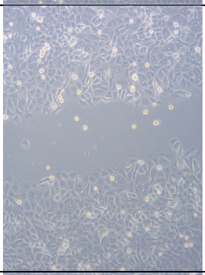	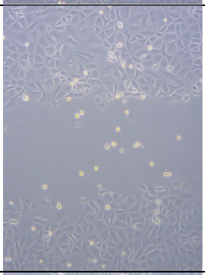
**24**	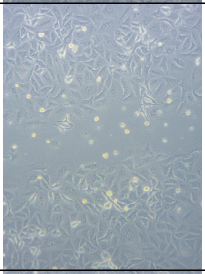	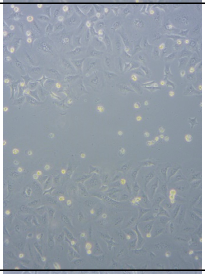	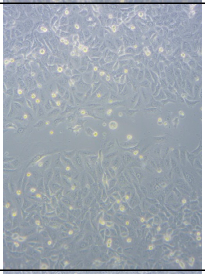	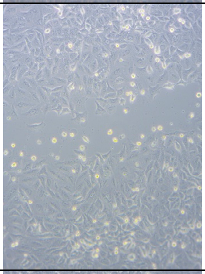
**35**	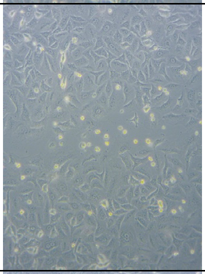	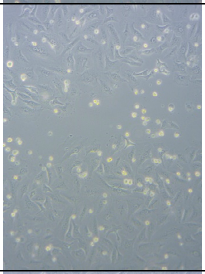	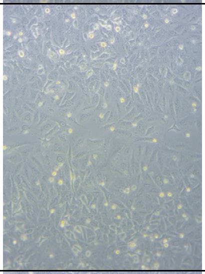	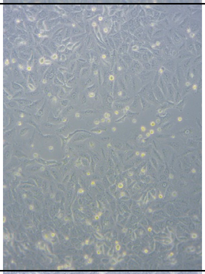
**56**	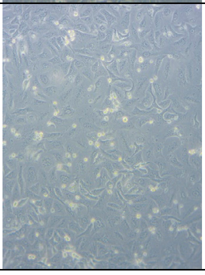	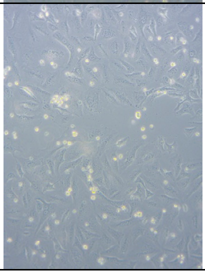	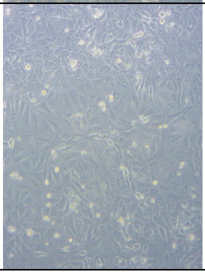	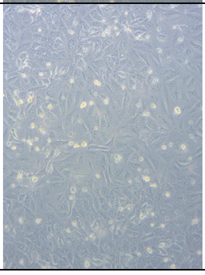

	**MCF7**		**A549**	
**Time [h]**	**shHtrA4**	**control**	**shHtrA4**	**control**
**0**	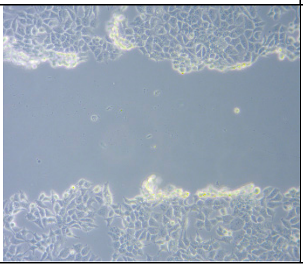	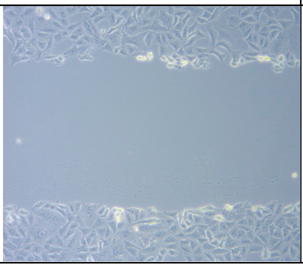	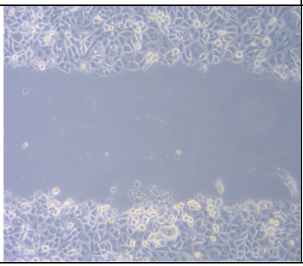	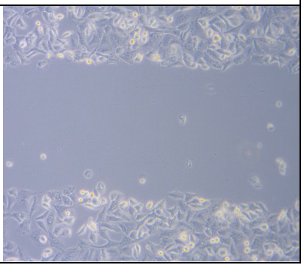

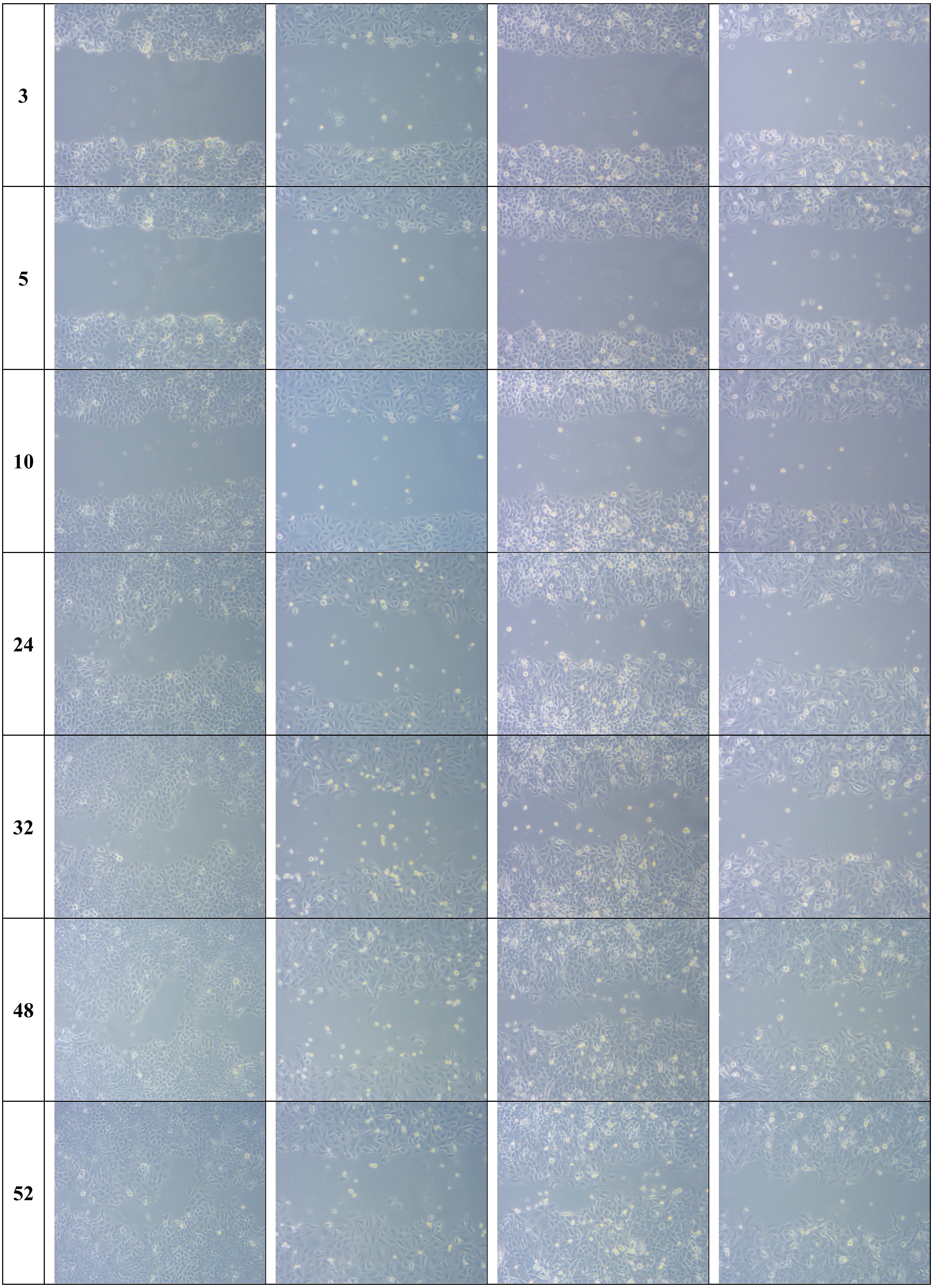
3
5
10
24
32
48
52

Figure S4. HtrA4 decreases motility of cancer cells. The motility of the A549 cells with the induced/not-induced exogenous production of the HtrA4, ΔN-HtrA4 or their proteolytically inactive variants (S326A) (A), and of the MCF7 and A549 cells with the *HtrA4* gene expression silenced/not silenced by shRNA (B) was analyzed using a wound healing assay. The analysis was performed in two independent approaches with at least ten photos. Representative images are shown. The quantitated data are presented in Figure 7.

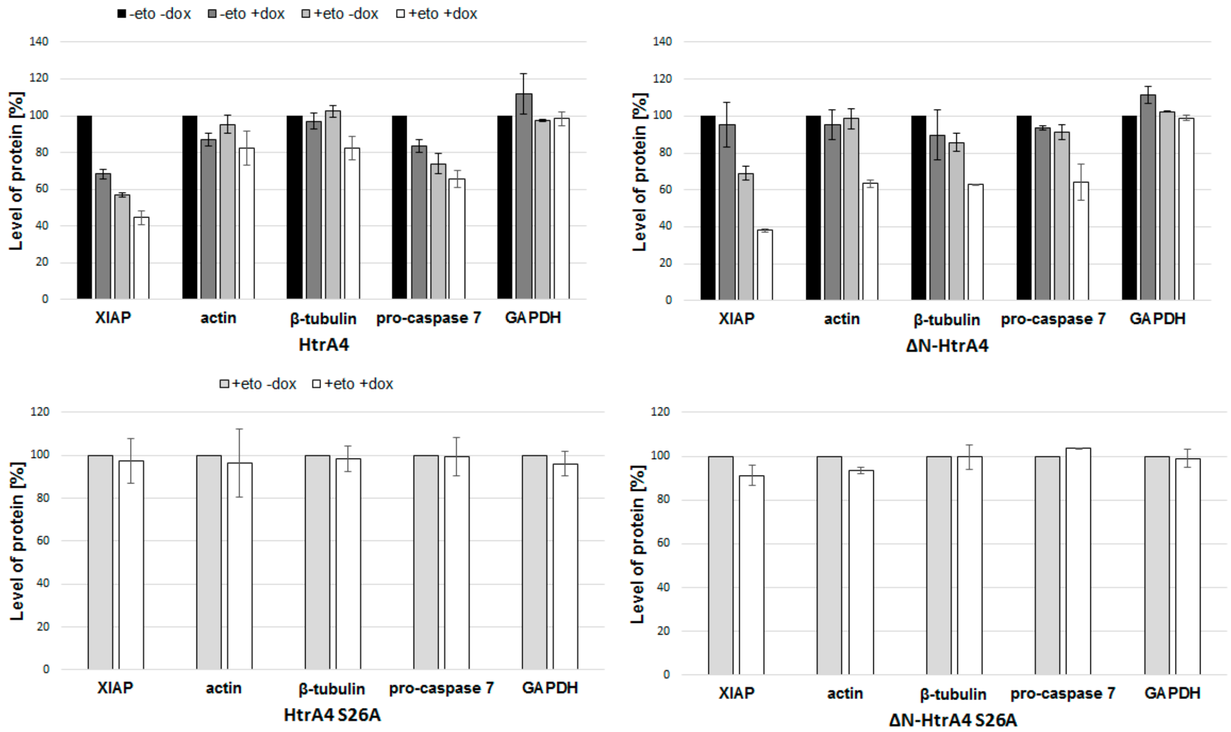

Figure S5. The levels of the tested proteins in A549 cells exogenously producing HtrA4, ΔN-HtrA4 and their proteolytically inactive variants in normal and apoptotic (induced by etoposide treatment) conditions. The data represent results of densitometric analysis of western blots (compare to Figure 9 of the main manuscript) and correspond to the mean ± SD of three independent experiments.

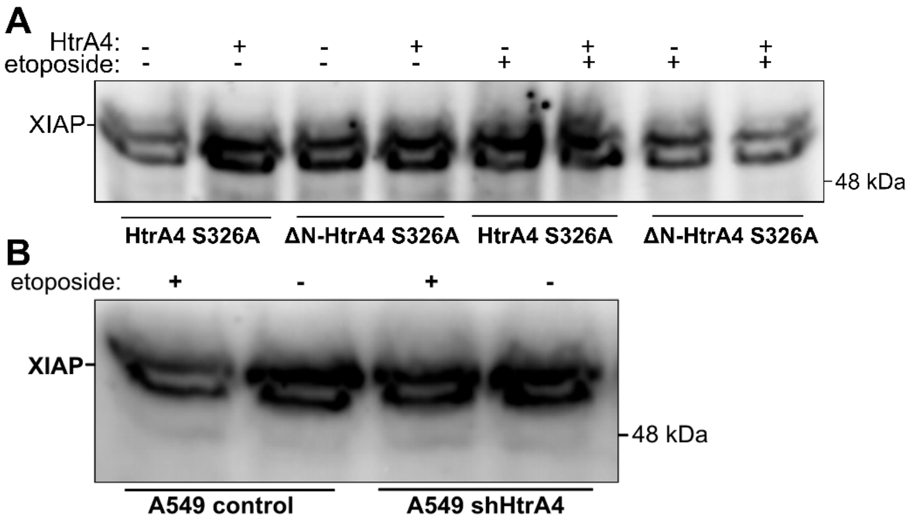

Figure S6. The XIAP levels in the A549 cells with exogenous expression of HtrA4/ΔN-HtrA4 S326A (proteolytically inactive variants) (**A**) and A549 cells with the *HtrA4* gene silenced by shRNA, under standard and apoptotic conditions induced by etoposide (**B**). The control cells were transduced with the empty pMKO.1 puro vector.
